# Current advances in niosomes applications for drug delivery and cancer treatment

**DOI:** 10.1016/j.mtbio.2023.100837

**Published:** 2023-10-21

**Authors:** Ali Moammeri, Masoumeh Mirzaei Chegeni, Hamidreza Sahrayi, Robabehbeygom Ghafelehbashi, Farkhondeh Memarzadeh, Afsoun Mansouri, Iman Akbarzadeh, Maryam Sadat Abtahi, Faranak Hejabi, Qun Ren

**Affiliations:** a School of Chemical Engineering, College of Engineering, University of Tehran, Tehran, Iran; b Department of Biology, Science and Research Branch, Islamic Azad University, Tehran, Iran; c Department of Chemical and Petrochemical Engineering, Sharif University of Technology, Tehran, Iran; d Department of Materials and Textile Engineering, College of Engineering, Razi University, Kermanshah, Iran; e School of Pharmacy and Pharmaceutical Sciences, Tehran Medical Sciences, Islamic Azad University, Tehran, Iran; f Department of Cell and Molecular Biology, Faculty of Biological Sciences, Kharazmi University, Tehran, Iran; g Laboratory for Biointerfaces, Empa, Swiss Federal Laboratories for Materials Science and Technology, 9014, St. Gallen, Switzerland

**Keywords:** Niosomes characterization, Niosome functionalization, Niosomes in gene delivery, Niosomes in cancer treatment

## Abstract

The advent of nanotechnology has led to an increased interest in nanocarriers as a drug delivery system that is efficient and safe. There have been many studies addressing nano-scale vesicular systems such as liposomes and niosome is a newer generation of vesicular nanocarriers. The niosomes provide a multilamellar carrier for lipophilic and hydrophilic bioactive substances in the self-assembled vesicle, which are composed of non-ionic surfactants in conjunction with cholesterol or other amphiphilic molecules. These non-ionic surfactant vesicles, simply known as niosomes, can be utilized in a wide variety of technological applications. As an alternative to liposomes, niosomes are considered more chemically and physically stable. The methods for preparing niosomes are more economic. Many reports have discussed niosomes in terms of their physicochemical properties and applications as drug delivery systems. As drug carriers, nano-sized niosomes expand the horizons of pharmacokinetics, decreasing toxicity, enhancing drug solvability and bioavailability. In this review, we review the components and fabrication methods of niosomes, as well as their functionalization, characterization, administration routes, and applications in cancer gene delivery, and natural product delivery. We also discuss the limitations and challenges in the development of niosomes, and provide the future perspective of niosomes.

## Graphical abstract

**Figure:**
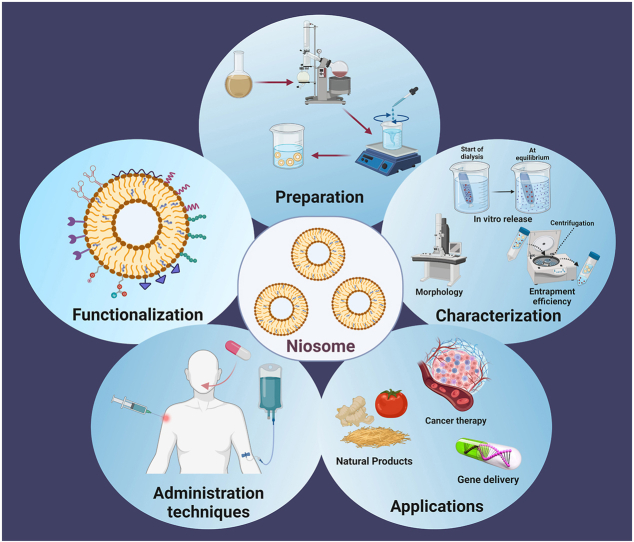

## 1 Introduction

The application of nanomedicine has fueled the development of nanocarriers. These nanocarriers can be loaded with different active pharmaceutical factors [1]. A major challenge faced by conventional drug delivery is unfavorable pharmacokinetics and distribution, which have the potential to cause unwanted side effects [2]. Two factors can reduce the effectiveness of drugs: degradation by the reticuloendothelial system and insufficient drug uptake at the target site. To overcome these challenges, nanocarriers have been extensively investigated in the past decades because they offer the following advantages: (a) facilitating targeted drug delivery to the disired site; (b) increasing surface area enhances absorption and bioavailability; (c) improving pharmacokinetics and biodistribution of therapeutic agents; (d) enhancing retention in biological systems and prolonging the efficacy of drugs [3]. Vesicular systems are one of the most innovative drug delivery systems which can offer an ideal approach for targeting, releasing, and controlling the delivery of therapeutic agents to the intended sites [4]. Niosomes are nanoscale spherical vesicles that can load a wide range of drugs within themselves. They are made of amphiphilic components that enable them to encapsulate both hydrophilic and hydrophobic drugs. These amphiphilic molecules make a bilayer as the membrane of vesicles that can help them be monolayer (having just one bilayer) or multilayer (having several bilayers and creating concentric spheres) based on the synthesis method. To have stable vesicles and improve other properties, some non-ionic surfactants, cholesterol, or their derivatives have been used in the synthesis [5]. Various molecules including amides, amino acids, alkyl ethers, alkyl esters, and fatty acids and surfactants such as alkyl esters (Tweens, Spans) and alkyl ethers (Brij) have been used to prepare niosomes [6]. As mentioned, niosomes can trap both hydrophilic and lipophilic drugs in their inner nuclei and outer bilayers. Thus, they can be used as carriers to deliver different drugs, hormones, and antigens [7,8].

Even though noisome shares high similarity with liposome, they are different in several aspects. On the one side, liposomes and niosome both are mono or multilayer spherical vesicles made by amphiphilic molecules. Their size is in the range of 10–1000 nm. They are biocompatible and can be used in drug delivery systems. On the other side, liposomes are composed of phospholipids, especially phosphatidylcholine, whereas niosomes are composed of non-ionic surfactants that make them chemically and physically more stable with prolonged durability. Furthermore, niosomes have other advantages like non-toxicity, easy and inexpensive fabrication method, and simple storage [[9], [10], [11]]. Normally, niosomes are stable at 25–37 °C, whereas liposome is stable only in a much narrow range of temperature, e.g. sometime instable even at room temperature [12]. Thus, as an alternative delivery system to liposomes, niosomes can eliminate the problems associated with large-scale production, sterilization, and storage associated with liposomes [13]. An optimal drug delivery system can be designed by varying the size, composition, surface load, number of lamellae, and drug entrapment efficiency [14,15]. Given the efficacy of niosomes as drug carriers in several clinical trials, the current focus is on obtaining the necessary licenses to apply niosomes as drug carriers [[16], [17], [18]]. Niosomes have been studied for delivering drugs to specific organs such as the brain and liver with improved pharmacokinetic properties [19,20]. Numerous publications and patents have been filed about niosomes in various fields, including pharmaceuticals, cosmetics, and food sciences. Researchers have investigated topical vaccine delivery using niosomes as carriers, which maintain the antigen in an aqueous core while enhancing penetration across the skin and initiating an immune response. Since niosomes are low in toxicity and capable of enhancing penetration, they are also studied for the ocular delivery of therapeutics. According to anticancer research, niosomes are capable of delivering anticancer agents more precisely and reducing their toxicity to reduce the severity of their side effects. It is of particular interest to use proniosomes for nebulizer drug delivery because they can deposit drug-loaded vesicles deep into the lung and improve therapeutic response [21]. This current study summarizes the latest applications of niosomes in delivery of natural products and genes, and cancer therapy. Their synthesis and characterization are aslo reviewed. Finally, the limitations and prospects of niosomes are discussed.

## 2 Components of niosomes

The niosome composition is a determinative factor in the fabrication, pharmacokinetic behavior, and application of drug-loaded niosomes. In general, a niosome comprises non-ionic surfactants, lipids such as cholesterol, charge-inducing agents, and hydration medium, which are relatively biocompatible and nontoxic [22].

### 2.1 Non-ionic surfactants

Non-ionic surface-active molecules are the fundamental elements in the preparation of niosomes. They are amphiphilic molecules with a polar head and a non-polar tail. These uncharged surfactants are more stable and less toxic than anionic, cationic, and amphoteric surfactants. These non-ionic surface-active agents, wetting agents, and emulsifiers have diverse capabilities including inhibiting p-glycoprotein, causing less hemolysis and irritation to cellular surfaces, enhancing permeability, and improving solubility [23]. Studies have reported the use of non-ionic surfactants in anticancer drugs [24], steroids [25], HIV protease inhibitors [26], and cardiovascular drugs [27] with improved uptake and targeting. Non-ionic surfactants with no charge on the polar head can be employed in the drug delivery carriers to offer controlled released rate, duration, and location [28]. HLB (hydrophilic-lipophilic balance), CPP (critical packing parameter), and gel liquid transition are important factors of entrapment efficiency (EE) [29]. Studies have indicated that a rise in the amount of HLB will increase the length of the alkyl chain and the size of the vesicle. It was reported that HLB values in the range of 14–17 are not suitable, while an HLB of 8 led to the highest EE [30]. For example, the EE of a lipophilic drug could be enhanced by utilizing a low-HLB surfactant [31,32]. The phase transition temperature is another influential factor in EE. A biodegradable surfactant such as Span 60 has a high transition temperature, offering high EE [33]. Surfactants with gel transfer temperatures below 10 °C can cause oxidation when combined with iodides, mercury salts, salicylates, sulfonamides, and tannins, phenolic substances [34].

### 2.2 Cholesterol

Cholesterol is not an essential additive in the formulation of niosomes, however, it can drastically affect the properties of niosomes if applied. It is common for niosomes to be formulated using cholesterol, for example in a 1:1 M ratio with a non-ionic surfactant [38]. Cholesterol can affect membrane permeability and stiffness, drug trapping efficiency, rehydration of dried niosomes, stability, storage condition, and toxicity [35], [36]. In addition to protecting the drugs from premature degradation, cholesterol also inhibits unwanted immunological and pharmacological effects. Nowroozi et al. [37] found that cholesterol affected niosome particle size dramatically. However, this impact is related with the type of non-ionic surfactant. A significant increase in cholesterol concentration from 20 to 40 % did not have a significant effect on particle size when Tween 60 was used as the non-ionic surfactant; wherease an increase in cholesterol caused a significant decrease in particle size when Brij 72 or Span 60 was used. Due to cholesterol's ability to enhance the hydrophobicity of bilayers [37], the surface free energy may be decreased resulting in a decrease in particle size.

A niosome containing cholesterol has a larger hydrodynamic diameter and is more effective at entrapping molecules. There are two general effects of cholesterol; on the one hand, cholesterol increases chain order in liquid-state bilayers, and on the other hand, cholesterol decreases chain order in gel-state bilayers. Cholesterol increases the rigidity of bilayers by decreasing the release rate of encapsulated material and, therefore, reducing the degradation rate. As a result of the charge, the interlamellar distance between successive bilayers increases in multilamellar vesicles, increasing the size of the entrapped volume in the end.

Cholesterol can also affect the vesicle structure of niosomes. Through hydrogen bonds formed between the hydroxyl groups of cholesterol and the alkyl chains of surfactant molecules, cholesterol enhances the stability of bilayers. Consequently, these interactions result in increased membrane cohesion and restriction of bilayer acyl chain movement. By influencing the fluidity of chains within bilayers, it increases the transition temperature of vesicles, thereby improving their stability [21].

### 2.3 Charge-inducing molecules

Charge-inducing molecules are used to stabilize the niosomes by electrostatic repulsion and help to prevent their fusion [40]. Dicetylphosphate (negatively charged), phosphatidic acid (negatively charged), and stearyl amine (positively charged) are among the charge-inducing molecules [38,41]. For example, Theansungnoen et al. used charge-inducing molecules to encapsulate two tryptophan-rich antibacterial peptides (KT2 and RT2) with niosomes [42].

### 2.4 Hydration medium

In addition to the components mentioned earlier, the fabrication of niosomes necessitates the use of a synthesis medium known as a hydration medium. Hydration is a crucial step in the production of niosomes, and phosphate buffer is commonly employed due to its ability to facilitate both niosome synthesis and drug loading [23]. The medium composition and hydration conditions such as pH, temperature, and time have an impact on the physicochemical properties of niosome nanoparticles, such as size, distribution, entrapment efficiency, and drug release profile. The pH level of the medium plays a pivotal role in both synthesis process and drug encapsulation. The applied pH of the buffer is determined by the solubility of the drug being encapsulated, and pH 7.4 has been found to yield stable vesicles with a small particle size when phosphate buffer is used [43]. Some investigations have demonstrated that the volume of the medium and the duration of hydration can also influence the final characteristics of drug-loaded niosomes, including entrapment efficiency and drug leakage [44]. It has been evident that longer hydration times result in reduced niosome size, higher entrapment efficiency, and greater stability, and more acidic media tend to lead to increased drug release [45,46].

## 3 Fabrication methods of niosomes

**Fig. 1:**
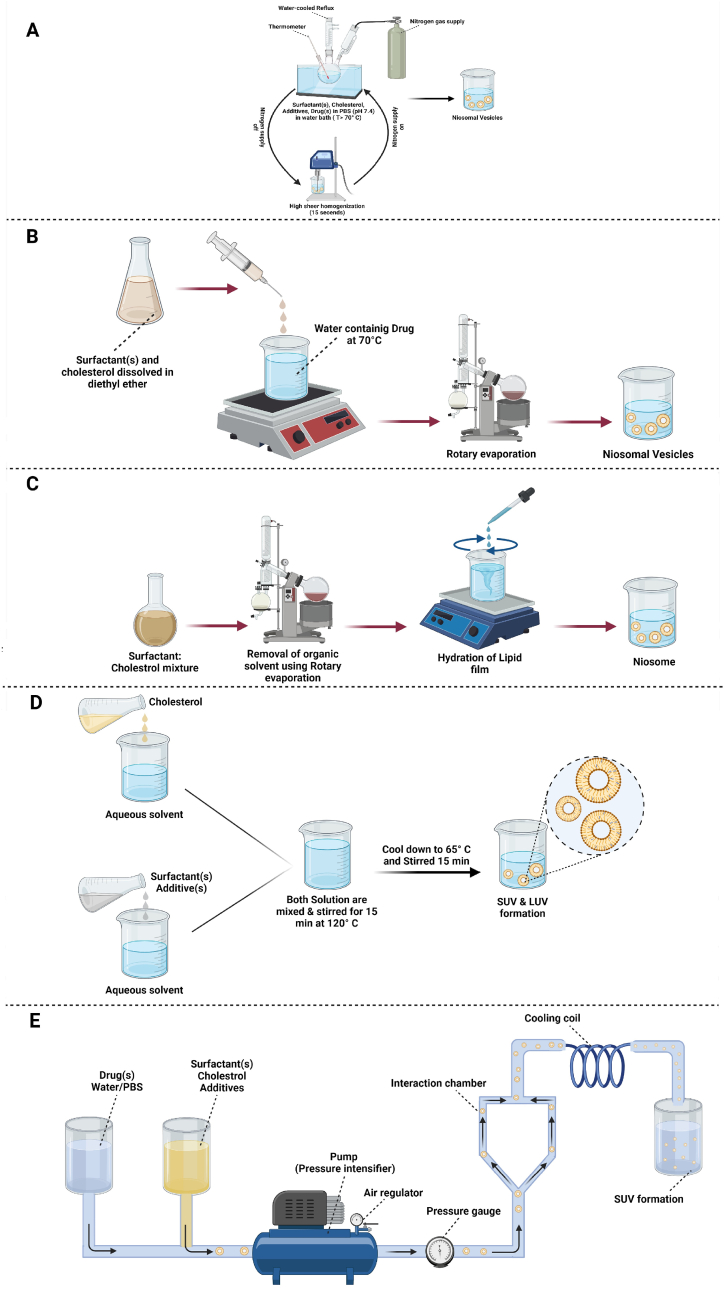

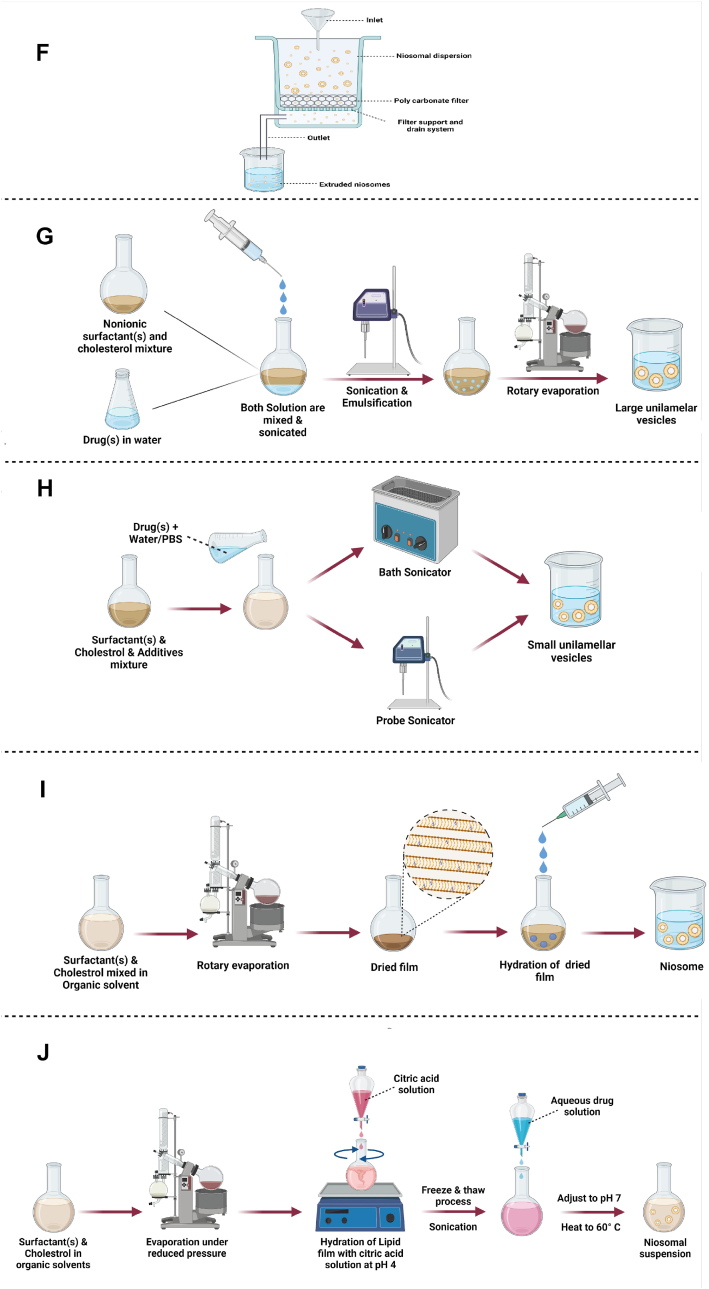
Schematic representation of different methods for preparations of niosome: (A) Bubble method, (B) Ether injection method, (C) Hand shaking method, (D) Heating method, (E) Microfluidization method, (F) Multiple membrane extrusion method, (G) Reverse phase evaporation method, (H) Sonication method, (I) Thin film hydration method, and (J) Transmembrane pH gradient method.

Various methods have been developed for synthesizing niosomes from ingredients mentioned in the previous section according to their particle size, lamellarity, and clinical applications. Some of these synthesis methods are illustrated in Fig. 1.

### 3.1 Bubble method

In this method, all the components are combined in three neck flasks at a given temperature. In an arranged system, one neck is located on a thermometer, another neck is used to purge nitrogen, and the last one is connected to a water-cooled reflux. All components are dispersed at 70 °C and homogenized for about 15 s. The mixture is instantly exposed to a stream of nitrogen gas. With this method, the synthesized vesicles are large and monolayer [47].

### 3.2 Ether injection

In this approach, cholesterol and surfactant are mixed in an organic solvent such as diethyl ether. The mixture is gently added into an aqueous drug solution at a constant temperature above 60 °C [48]. Single-layer vesicles of the surfactant-containing drug with variable diameters of 50–1000 μm are then formed upon solvent evaporation [49].

### 3.3 Manual shaking method

This method is similar to the thin-layer hydration method described below. Here, surfactants, cholesterol, and other lipophilic additives are dissolved in an organic solvent, and evaporation of the organic solvent leads to the formation of a thin layer. The milky mixture containing the niosomes is then formed after hydration of the thin layer and gentle mechanical shaking [50].

### 3.4 Heating method

Surfactants, cholesterol, and other additives are separately hydrated in a buffer solution under a nitrogen atmosphere. The glass containing cholesterol is heated to about 120 °C for 15–20 min and cooled to 60 °C. The other ingredients are then added to the stirring cholesterol container for 15 min. The prepared niosomes are placed at room temperature for 30 min and stored in a refrigerator (at a temperature of 4–5 °C) under an N_2_ atmosphere to stabilize them [51].

### 3.5 Microfluidization method

In this method, drugs and surfactants are dissolved in a solvent and pumped under pressure from a reservoir to an interaction chamber packed with ice. The solution is passed through a cooling loop to absorb the heat generated during the process. This method can yield niosomes of smaller size with excellent uniformity [49,52].

### 3.6 Multiple membrane extrusion methods

This method is suitable for controlling the size of a niosomal formulation. A mixture of surfactant, cholesterol, and diacetyl phosphate in chloroform is converted into a thin film by evaporation. The resulting film is hydrated with an aqueous drug solution, and the suspension is extruded through polycarbonate membranes [53].

### 3.7 Reverse phase evaporation method

Surfactants and cholesterol are combined in an organic solvent, then an aqueous solution is added to the organic phase. The two-phase system is homogenized, and the organic phase is removed under negative pressure. Subsequently, large monolayer vesicles can be obtained [53].

### 3.8 Sonication method

First, the drug-containing buffer solution (e.g. rifampicin and ceftriaxone sodium) is added to a mixture of cholesterol and surfactants (e.g. Span 60, Pluronic L121, and Dicetylphosphate) in a glass vial. Next, the mixture is probe-sonicated at 60 °C for 3 min by a sonicator with a titanium probe to yield niosomes. Multilamellar vesicles (MLVs) are made, and unilamellar vesicles are also achievable [54].

### 3.9 Thin film hydration method

Surfactants, cholesterol, and other lipophilic additives are dissolved in an organic solvent within a round bottom flask. A rotary vacuum evaporator is used to remove the organic solvent. Afterward, organic solvent-soluble materials form a thin, dry layer on the inner surface of the flask. Water or an aqueous solvent containing the drug is added to the flask at temperatures above the transfer temperature, i.e., the temperature required to hydrate the thin layer. Multilayer vesicles are formed during hydration. Appropriately cut-off-sized membranes or high-pressure homogenizers can be used to produce small-size niosomes [39,52,55].

### 3.10 Transmembrane pH gradient method

Niosomes can be formed by varying pH from the core to the outer membrane. Surfactants and cholesterol are dissolved in an organic solvent. A thin film is then created after the solvent evaporation, which is subsequently hydrated by the acidic solution, and then the product is frozen. A buffer with a neutral pH (7.0) is added to niosomes, including an aqueous drug solution to maintain the pH. Weakly acidic drugs (normally with pKa <5) can be ionized by changing pH from the outer membrane to the core [56]. The advantages and disadvantages of the mentioned niosome preparation techniques are summarized in Table 1. According to the conducted works on using niosomes in the delivery of miscellaneous therapeutics, researchers have always been interested in incorporating a functionalization step into fabrication techniques to provide an efficient niosomal carrier.

**Table 1:** The advantages and disadvantages of techniques used for niosome preparation.

Method	Advantages	Disadvantages	The average size of the particles	Ref
Bubble method	Easy to implement, no organic solvents	Instability for long-term usage	–	[57]
Ether injection	Simple and easy process, size controllable	Heterogeneous and large PDI, low EE, toxicity due to residual organic solvent	393.9 nm	[58]
Hand shaking method	Large diameter, multi-lamellar niosome	Large in size	100–140 nm	[59]
Heating method	No organic solvent	Not suitable for heat sensitive drug	–	[60]
Microfluidization method	Improved uniformity, small vesicles possible, adequate reproducibility	Membrane impurity, need of organic solvent, sensitive to hydrolysis and/or oxidation, tendency to agglomerate and/or fusion, leakage of encapsulated drug	157 nm	[61]
Multiple membrane extrusion method	Controllable size, small unilamellar vesicles	Not appropriate for heat-unstable drugs	–	[62]
Reverse phase evaporation	Single layer, great encapsulation efficiency, capable of encapsulation of small & big molecules	Macromolecule contamination, solvent or sonication needed, toxicity because of residual organic solvent	–	[62]
Sonication method	Simple and easy process, controllable particle size, no organic solvents needed, green method	Potential titanium probe loss because of the high temperature, high energy consumption	100–140 nm	[21]
Thin film hydration	Low PDI, high stability, suitable for scale-up, good bimolecular film formation	Multilamellar and big-size, low EE	388 nm	[63]
Transmembrane pH gradient method	Easy and simple method, high EE of weak amphiphilic and acidic drugs	High PDI, low reproducibility with difficulty in standardization, organic solvent needed		[64]

## 4 Functionalization of niosomes

**Fig. 2:**
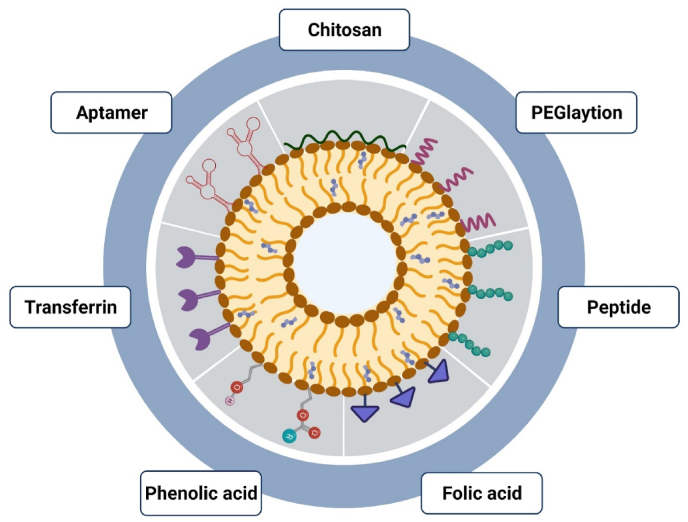
Functionalization of niosome with chitosan, aptamer, transferrin, phenolic acid, folic acid, peptide, and by PEGylation.

Following the development of nanotechnology, the use of nanoparticles as drug carriers increased the efficiency and safety of the delivery of drugs to the target site. Nowadays, the conjugation of biomolecules on the surface of these drug nanocarriers can help to precisely target and concentrate drugs in the desired location. Certain surface ligands can bind or be adsorbed on niosome nanoparticles to promote targeted drug delivery to the intended receptor on the cell. Here, modification of niosomes by the surface ligands is surveyed for their effect on niosome structure, and summarized in Table 2 and Fig. 2. The most-used functionalizing biomolecules in niosome-assisted drug delivery, including aptamer, peptide, transferrin, folic acid, chitosan, and phenolic acid, are provided as follows.

**Table 2:** List of niosomal formulations functionalized with various agents. EE: encapsulation efficiency.

Targeted Ligand	Drug	Formulation	Particle size (nm)	Zeta potential (mV)	PDI	EE (%)	Outcome	Ref
Aptamer	Doxorubicin (Dox)	PEGNIO/Dox	152.7 ± 34	− 3.56 ± 0.27	0.214	39.52 ± 1.8	Cytotoxicity tested for HeLa and U87 cells overexpressing MUC1	[69]
		PEGNIO/Dox/CysTAT–MUC1	164.5 ± 40	− 8.62 ± 0.50	0.275			
Aptamer	Ru (III)-complex HoThyRu	Niosome_HoThyRu	56.8 ± 0.1		0.32 ± 0.02		The bioactive effect of Ru (III) increased with AS1411 and anti-proliferative activity reported on HeLa cells.	[70]
		AS1411/Niosome_HoThyRu	86.7 ± 0.6		0.32 ± 0.04			
Peptide	Dox and Curcumin	PEGNIO/D–C	144.1 ± 61		0.152	D:23.3 ± 1.6, C:32.6 ± 1.9	Synergistic effect of drugs with functionalized nanoparticles reported. Cytotoxicity and anti-proliferative effect tested for U87 cells.	[72]
		PEGNIO/D–C/tLyp-1	146.1 ± 69		0.140	D:22.0 ± 1.5, C:31.2 ± 1.8		
Peptide	Tenofovir	PEG-NI	154.8 ± 2.6	− 5.85	0.262 ± 0.032	71.00 ± 0.01	The anti-HIV effects of PEGylated niosome were superior to those conjugated to TAT systems.	[74]
		TAT-NI1	208.9		0.39	75		
Transferrin	HCPT (10-Hydroxycamptothecin)	PEG-NS	97 ± 8	−3.76 ± 0.02	0.182 ± 0.042	93.59 ± 0.13	HCPT loading by PEGylated and transferrin-functionalized niosomes showed significant toxicity in KB, K562 and especially S180 cells.	[80]
		Tf-PEG-NS	116 ± 9	−3.44 ± 0.03	0.222 ± 0.021	93.00 ± 0.38		
Transferrin	Dox	L64ox/Chol-D	350 ± 11		0.243	37.3 ± 0.5	The formulation showed dose-dependent toxicity in MCF-7 and MDA-Mb-231 cell lines.	[76]
		L64ox/Chol-D-Tf	361 ± 11		0.267	37.0 ± 0.5		
FA	Curcumin	Fe3O4@PLGA-PEG	164.2 ± 2.1		0.163		The optimal formulation induced apoptosis in Hela229 cells and showed good drug loading and release.	[90]
		Fe3O4@PLGA-PEG@FA	190.4 ± 5.3		0.112			
Chitosan	Ursolic Acid	Nio-UA	198.7 ± 13.8	−57.5 ± 11.9	0.29 ± 0.02		Cytotoxicity increased by adding chitosan layers in Hela cells, while less sensitive in Huh7it cells.	[84]
		Nio-UA-CS	237.7 ± 6.2	3.88 ± 1.5	0.33 ± 0.03			
Phenolic Acids	Curcumin	T80C	201.25 ± 9.32	−27.8 ± 0.28	0.251 ± 0.036	14.6 ± 3.15	The synergistic effect of Curcomin with GA and CF showed better antioxidant activity.	[89]
		T80C-GA	91.70 ± 4.50	−13.3 ± 1.0	0.225 ± 0.042			
		T80C-CF	83.70 ± 2.60	−14.9 ± 0.85	0.273 ± 0.007			
		T80C-FR	72.5 ± 1.11	−15.9 ± 0.40	0.281 ± 0.018

### 4.1 Aptamer

Aptamers are three-dimensional folded structures composed of single-stranded DNA or RNA molecules, typically ranging from 20 to 100 nucleotides in length [65,66]. They can bind to a molecular target specifically. Their remarkable attributes, including high binding affinity and specificity for cell membrane receptors, make them highly suitable ligands for targeted drug delivery systems [67,68]. Seleci et al. have synthesized a drug delivery platform based on PEGylated niosome (PEGNIO) that is modified with cell-penetrating peptide (CysTAT) and cell-specific aptamer (S2.2), which targets MUC1 glycoprotein overexpressing in many cancer cells. Doxorubicin (Dox) was encapsulated in this platform and the cellular uptake, intracellular distribution, and *in-vitro*, cytotoxicity studies were carried out on MUC1-positive HeLa and MUC1-negative U87 cells. After aptamer conjugation, the zeta potential of the niosomal platform was reduced. The result showed a high level of Dox in MUC1-positive Hela cells after treatment with PEGNIO/DOX/CysTAT–MUC1 conjugate in comparison to free Dox and non-targeted niosome, but in MUC1-negative U87 free Dox showed higher uptake. The *in-vitro* cytotoxicity assay showed superior cytotoxicity of MUC1-targeted niosome in MUC1-positive Hela cells to free Dox and non-targeted niosome, whereas not in the MUC-1-negative U87. A CysTAT-modified cell-penetrating peptide was conjugated to the amine group of MUC1 aptamer, using bis(sulfosuccinimidyl) substrate (BS3) as a crosslinking agent. By forming a thioether linkage, CysTAT-MUC1 conjugate was attached to PEGNIO encapsulated in Dox (PEGNIO/Dox) [69]. Riccardi et al. have examined the effect of AS1114 aptamer, as a targeting agent and Ru (III)-complex HoThyRu as an anticancer drug in HeLa cervical cancer cell line and HCC2998 and HTB-38 colorectal cancer cell lines. This aptamer can be used to target nucleolin, a compelling protein that is overexpressed in cancer cells. The optimal and satisfactory formulation of AS1411/niosome with the desired drug was prepared.. The AS1411/niosome_HoThyRu showed high bioactivity and cytotoxicity on Hela cells in comparison to niosome_HoThyRu and free drug, but colorectal cancer cell lines did not show remarkable deference. A set of functionalized nanosystems was obtained by adjusting the charge ratio between (number of anionic phosphate groups) oligonucleotides and (number of cationic amino groups) cationic lipids [70].

### 4.2 Peptide

Surface modification of the niosome with peptides can be actively used for targeted drug delivery through interaction with cell surface receptors and drug penetration through endocytosis [71]. In a study by Seleci et al., PEGylated niosome (PEGNIO) was prepared for the multi-delivery of Dox (D) and curcumin (C). The tumor homing and penetrating peptide (tLyp-1) was conjugated to a co-loaded niosome (PEGNIO/D–C). A higher uptake of PEGNIO/D–C/tLyp-1 than PEGNIO/D–C was observed for U87 cells, which is related to the ability of tLyp-1 to home in and penetrate selectively into tumor cells overexpressing NRP-1 receptor. Moreover, the cellular uptake of targeted and non-targeted nanosystems has no difference in hMSC cells. PEGNIO/D–C/tLyp-1 showed the highest cytotoxicity compared to PEGNIO/D–C and free D–C for U87 cells due to the conjugation of the targeting ligand [72]. Similarly, in anti-glioma treatment, Seleci et al. used PEGNIO-tLyp-1 encapsulated topotecan (TPT) as a targeted nanoparticular drug delivery system fabricated via microfluidics. The hydrodynamic diameter of TPT-loaded niosomes was increased after the conjugation of tLyp-1. The binding of tLyp-1 peptide to NRP-1 which is overexpressed on U87 cells causes the internalization of the nano platform and leads to higher cytotoxicity of PEGNIO/TPT/tLyp-1 in comparison to PEGNIO/TPT and free TPT in glioma cells [73]. The tLyp-1-targeted niosomes were prepared by forming a thioether link between the thiol group of tLyp-1 and the maleimide terminal group of the PEG chains [73]. Yadavar-Nikravesh et al. investigated the anti-HIV effect of PEGNIO loaded with an anti-HIV drug (Tenofovir) modified with TAT peptide on the HIV-infected HeLa cells. Eventually, these modified niosomes showed toxicity and antiproliferative effects against HIV-1 [74].

### 4.3 Transferrin

Transferrins (TFs) are known as glycoproteins responsible for transporting iron ions. Moreover, transferrin receptors on cells contribute to receptor-mediated endocytosis. TFs are overexpressed in cancer cells. As a result, transferrin conjugation with drug nanocarriers enhances their selectivity to tumor cells, leading to greater efficacy in drug-resistant cells [75]. In this way, Tavano et al. prepared a tumor-targeted niosomal system based on transferrin as a ligand for the delivery of Dox. The cytotoxicity and cellular uptake of TF-conjugate niosomes were evaluated on MCF-7 and MDA-MB-23. There was no significant difference in particle size after the conjugation of TF to niosomal formulation. TF-conjugate niosomes indicated higher cellular uptake and cytotoxicity than non-targeted formulation on MCF-7 and MDA-MB-23 cell lines [76]. Also, niosome-conjugated TF for controlled release of Dox and curcumin was prepared to attach to the specific TF receptors on breast cancer cell lines and internalize through the endocytosis pathway [77]. Targeted niosomal formulation considerably improved the cellular uptake into MCF-7 and MDA-MB-231 cell lines compared to non-targeted niosomes [78]. Seleci et al. developed multifunctional TF-decorated niosomes combining magnetic iron oxide nanoparticles (MIONs) and quantum dots (QDs) for the imaging of glioma. An enhancement in cellular uptake was found after the decoration of TF. The negative contrast effect was enhanced and the fluorescence intensity improved under fluorescence microscopy [79]. In a study by Hong et al., the antitumor effects of TF-modified PEGylated niosomes (TF-PEGNIO) for delivery of hydroxycamptothecin (HCPT) were assessed on cancer cell lines, especially S180 [80]. The drug release rate of TF-PEGNIO showed a faster two-phase pattern compared to the non-modified formulation. The cytotoxicity results showed that the conjugation of TF with PEGNIO could be a promising approach in targeted tumor treatment [80].

### 4.4 Folic acid

Folic acid (FA), known as vitamin B6, tends to bind to folate receptors (FR), which are overexpressed in some solid tumors, thus it could be an efficiently targeted ligand for cancer treatment. In a study by You et al., the antiproliferative properties of FA-conjugated PEGNIOylated niosomes loaded with curcumin were assessed on the cervical cancer cell line. The conjugation of FA into the niosomal formulation increased the size of the vesicles but with acceptable PDI. The result of cellular uptake demonstrates that FA-conjugated niosome internalizes curcumin by FA-receptor-medicated endocytosis and improves the anti-tumor effect on HeLa229 cells compared to non-targeted formulation [81]. Another FA-functionalized niosome was prepared for delivery of curcumin and letrozole for chemotherapy of breast cancer. FA-functionalized niosome showed good biocompatibility on HEK-293 normal cells and cytotoxicity effects on breast cancer cell lines. Also, targeted-niosome enhanced apoptosis rate against MCF-7 cells and MD-MB-231 cell lines due to higher cellular uptake through folate receptor-mediated endocytosis in comparison to free drug and non-targeted formulation [82]. Honarvari et al. formulated a PEGylated niosome decorated with folic acid for the delivery of curcumin for breast cancer therapy. PEG-FA-modified niosomes showed more cellular uptake in MCF7 and 4T1 cell lines than free drugs and non-modified niosomes. Also, the gene expression level of Bcl2 was lowest for PEG-FA@Nio-Curcumin compared to free drug and Nio-Curcumin which indicates the promising ability of this platform for breast cancer therapy [83].

### 4.5 Chitosan

Chitosan as a natural linear polysaccharide is a substance that increases cell absorption and has received a great deal of attention in medical science [233]. A study by Miatmoko et al. showed that the addition of chitosan to the ursolic acid (UA) loaded niosome could increase cellular uptake by Hela cells which is related to clathrin-mediated endocytosis transporting UA niosomes into the cells [84]. The chitosan layers enhance cytotoxicity effects on Hela cells in comparison with niosomal formulation without chitosan [84]. An optimum formulation of niosome adorning with chitosan (CS) for co-delivery of Dox and vincristine (VIN) was prepared for breast cancer therapy. Coated niosome (DOX + VIN/Nio/CS) showed lower IC50 on the SKBR3 cell line in comparison to non-coated niosome (DOX + VIN/Nio) [85]. Wiranowska et al. assessed the intracellular and extracellular localization of a targeted drug delivery system based on paclitaxel (PTX)-encapsulated niosome embedded in a chitosan hydrogel that has an affinity to MUC1 that overexpressed on OV2008 cells. The result indicates the high fluorescence intensity of chitosan–niosome–PTX near the OV2008 cell surface compared to a normal IMCC3 cell surface. Also, intracellular fluorescence intensity was 2 times higher than in normal IMCC3 cells [86].

### 4.6 Phenolic acid

Phenolic acids refer to the phenolic compounds that have a carboxylic acid group and possess high *in-vitro* antioxidant activity [87]. Phenolic acids could balance a healthy redox due to their ability to inhibit or delay undesired oxidative degradation [88]. In a study by Mazzotta et al. phenolic acids (gallic (GA), ferulic (FR), and caffeic acid (CF)) were conjugated to the surface of niosome and the antioxidant activity of different niosomal formulations were evaluated. It was found that the conjugation of phenolic acids to the surface of niosomes reduced the size of the vesicle, due to the formation of hydrogen bonds and increased vesicle cohesion after attachment. Also, Nio-GA was smaller than Nio-FR and Nio-CF due to its more hydrophobic properties. This property accelerated the drug release after loading curcumin into Nio-GA compared to Nio-FR and Nio-CF. Examination of antioxidant activity showed higher antioxidant activity of GA or FR-functionalized niosome than CF-functionalized formulation [89].

Researchers have shown that phenolic compounds form intermolecular hydrogen bonds with lipid bilayers that lead to higher membrane cohesion and, consequently, reduced vesicle size [89]. By increasing hydrophobic attraction forces among surfactant head groups, phenolic acid/surfactant interactions led to a small surface area for molecules and a compact structure [89].

## 5 Types of niosomes

**Fig. 3:**
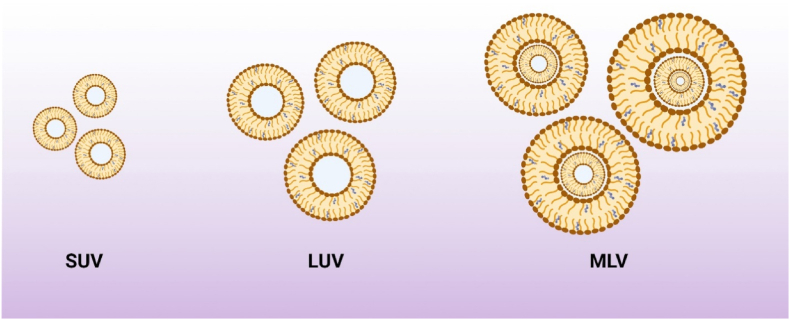
Types of niosomes based on the size and number of lamellar. SUV: small unilamellar vesicles, LUV: large unilamellar vesicles, MLV: multilamellar vesicles.

Depending on the size and number of layers, niosomes can be grouped into small unilamellar vesicles (SUV), large unilamellar vesicles (LUV), and multilamellar vesicles (MLV) (Fig. 3) [91]. The size of SUVs varies from 10 to 100 nm; they can be synthesized using sonication, high-pressure extrusion, and high-shear homogenization [92]. SUVs are thermodynamically unstable and tend to aggregate; they also offer low entrapment efficiency for hydrophilic agents [92]. LUVs have a diameter of 100–1000 nm with relatively high water-to-surfactant ratios, which can be prepared by the transmembrane pH gradient method (remote loading), reverse phase evaporation, solvent injection, heating, dehydration, and rehydration methods. Thanks to the minor usage of non-ionic surfactants, LUVs are an efficient option for large-scale production [93]. The size of MLVs varies from 0.5 to 10 µm; they have different bilayers enclosing the aqueous medium individually. MLVs are the most used niosomal carriers with proper mechanical stability ideal for embedding lipophilic bioactive compounds through a facile preparation technique. The particle size is a fundamental characteristic of niosomes playing a crucial role in determining the pharmacodynamic and pharmacokinetic parameters of loaded drugs [94]. Different characterization techniques have been applied to determine the morphological and physiochemical features of niosomes which are examined as follows.

## 6 Characterization of niosomes

**Fig. 4:**
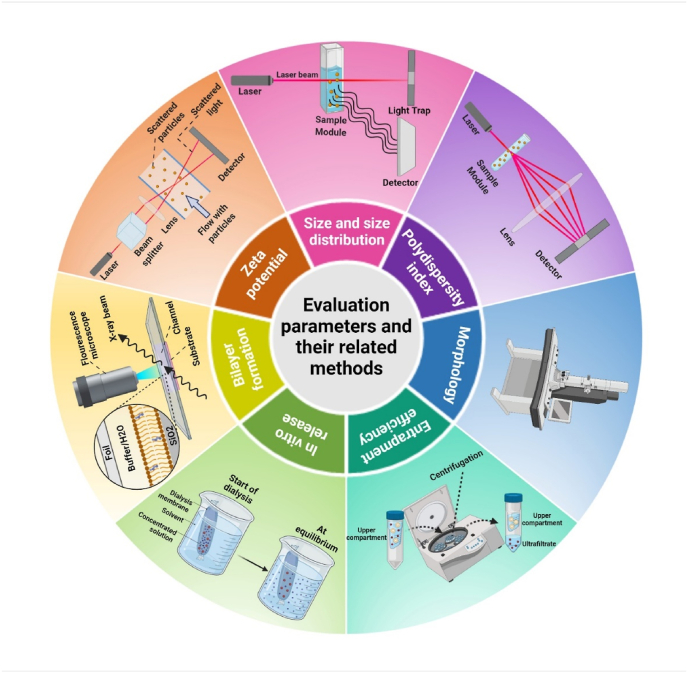
Different methods and techniques used for niosome characterization and evaluation. (1) Dynamic light scattering (DLS) used for size and zeta potential analysis, (2) DLS used also for the analysis of polydispersity index (PDI), (3) Scanning electron microscopy (SEM) and transmission electron microscopy (TEM) used for morphology study, (4) filtration-centrifugation method for study of entrapment efficiency, (5) dialysis method for study of in-vitro drug release, and (6) X-ray diffraction (XRD) for surface characterization.

Characteristics of niosomes, including size, distribution, zeta potential, morphology, EE, and release behavior, can be studied by various analyses [95]. Particle size is a critical factor for niosomes as it provides information about the physical properties and stability of a niosomal formulation [23]. Different techniques such as light microscopy, electron microscopic analysis, SEM (scanning electron microscope) [96], TEM (transmission electron microscope) [97], freeze-fracture replicator, dynamic light scattering (DLS), and zeta-sizer [98], have been used to characterize the size and morphology of niosomal formulation [99]. DLS is a photon correlation spectroscopy that can be used to assess particle size (range of 3–3000 nm). In this technique, the laser beam is scattered by the niosomes. A fixed or inconstant scattering angle is observed as a function of time accompanied by the intensity of scattered light fluctuations due to the collision of particles caused by random Brownian motion [100]. Smaller particles produce higher fluctuations due to their higher diffusion coefficient, whereas larger particles move relatively slowly and cause fewer changes [101]. The PDI is defined as a distribution of niosome size. Niosomal formulation with PDI values below 0.5 shows a monodispersed sample. DLS measurement method is often combined with microscopic techniques to achieve reliable results [102]. Microscopic techniques are utilized to analyze the morphology of niosomes. TEM, negative staining transmission electron microscopy (NS-TEM), and freezing fracture transmission electron microscopy (FFTEM) are preferentially used for liquid samples while SEM is often utilized for solid samples. Atomic force microscopy (AFM) and scanning tunnel microscopy (STM) can be also applied to assess the characteristics of nanostructures and the thickness of two layers of niosomes due to their analytical capability in the upright axis [103]. Evaluation parameters and their related methods, including zeta potential, formation bilayer, *in-vitro* release, entrapment efficiency, morphology, PDI, and size are depicted in Fig. 4.

### 6.1 Encapsulation efficiency (EE)

EE is defined as the ratio of drug molecules encapsulated into the niosomes nanoparticles to the total used drug, and can be determined by the following equation:

EE = (Amount of trapped drug/Total amount of initially added-drug) × 100 %

The unencapsulated drug molecules can be separated from the trapped ones using dialysis, filtration, gel chromatography, or centrifugation methods. Spectrophotometry and gel electrophoresis can be employed for determine the loaded drugs [104]. Also, UV densitometry is applied for genetic materials and fluorescence markers are applicable for the biomarkers [105].

### 6.2 Zeta potential

The charges in niosomes can be detected based on zeta potential measured with a zeta potential analyzer, nano zeta-sizer, microelectrophoresis, pH-sensitive fluorophores, high-performance capillary electrophoresis, and DLS instrument. Zeta potential determines the physical stability of niosomes. Surface potential can be calculated by laser. Niosomes with zeta potentials over +30 mV or lower than −30 mV have adequate strength [106]. Niosomes are charged and electrostatic repulsion maintains their stability by avoiding agglomeration and interfusion [107].

### 6.3 Bilayer formation

Niosomes could be found in single-layer or multi-layer forms [108]. Small-angle X-ray scattering (SAXS), nuclear magnetic resonance spectroscopy (NMR), and AFM are used to characterize the number of lamellae. SAXS and energy-dispersive X-ray diffraction (EDXD) can also detect the thickness of the niosomal bilayer [19,38]. Niosomal membrane liquidity enables the membrane to distort without altering the unity of the bilayer. Niosomal membrane liquidity and microviscosity can be respectively measured by the movability of a fluorescent probe and fluorescent polarization to investigate the packaging structure of lipid bilayers [94,[109], [110], [111]].

### 6.4 *In-vitro* release

*In-vitro* drug release can be investigated by dialysis membranes. This parameter is under the influence of many factors, including drug concentration, hydration volume, membrane type, and niosome composition [112]. To test *in-vitro* drug release, the drug-containing niosomal suspension is placed into a dialysis bag, closed at both ends, and placed in a breaker of phosphate saline buffer (PBS) at a fixed temperature using a magnetic stirrer. The medium is sampled at predefined intervals and substituted with an equal volume of the fresh medium. Samples are analyzed by suitable assessment to determine the concentration of the released drug [113]. Another alternative is to place a dialysis membrane between the donor and the recipient by Franz diffusion cells. In this method, the niosomal suspension is loaded onto the donor. The receiver is placed in PBS (pH = 7.4) at 37 °C. Samples are assembled from the receiver compartment at regular intervals and substituted with an equal-release medium [114].

## 7 Application of niosomes

### 7.1 Delivery of phytochemicals

Natural products have been utilized for their therapeutic effects for centuries. The high cost of developing new drugs has raised research interest in the delivery of plant constituents and natural products as new pharmaceutical agents [54,115]. Natural products, also known as phytochemicals, can be found and extracted from plants, especially vegetables, fruits, and grains. These phytochemicals have amazing medicinal features such as anti-cancer, antioxidant, antimicrobial, and anti-inflammatory activities. However, most of them cannot be administered directly because of some limitations like poor solubility or instability, requiring novel delivery approaches such as encapsulation using niosome nanoparticles [[116], [117], [118]]. Various medicinal plants are used, but some are more effective and popular, such as curcumin, resveratrol, rice bran, lycopene, ginger, and ellagic acid (EA).

Curcumin (1,7-bis-(4-hydroxy-3-methoxyphenyl)-1,6-Heptadiene-3,5-dione) is produced by Curcuma plants and is one of the most valuable pharmaceutical agents with effective characteristics against different cancers such as breast, prostate, lung, and bone cancers [29]. It also exhibites antioxidant, antiviral, and anti-inflammatory benefits besides anti-cancer activities without side effects [119]. Its natural hydrophobicity leads to its poor water solubility and low absorption, seriously limiting its bioavailability. These drawbacks can be resolved by encapsulation of curcumin molecules into the lipidic bilayers of niosome vesicles. Sharma et al. encapsulated curcumin along with doxorubicin (Dox) into the niosome nanoparticles to achieve hydrophobic and hydrophilic formulation as a multi-delivery system for cancer therapy [120]. Naderinezhad et al. [119] and Alemi et al. [121] formulated niosomal encapsulated curcumin and PTX to explore their synergistic effects against breast tumors. Kumar et al. prepared a gel formulation containing niosomal curcumin encapsulations for transdermal delivery targeting high-efficacy anti-inflammatory and anti-arthritic activities [122].

Resveratrol is mostly found in the skin of red grapes and blueberries. It is a type of natural phenol with effective pharmaceutical features due to its antioxidant, anti-inflammatory, cardioprotection, platelet aggregation inhibition, and anti-cancer activities [123]. However, its properties of low hydrophilicity, fast oxidation, weak bioavailability, and light sensitivity have resulted in its quick isomerization to the inactive state [123]. Several researchers considered niosomal entrapment to protect resveratrol and enhance its delivery. Pando et al. formulated niosomal resveratrol encapsulation using a modified film hydration method for oral administration [124]. They also investigated different preparation methods for its topical delivery [125]. Machado et al. explored niosomal resveratrol incorporation into the gelatin-based hydrogel for tunable delivery [123].

Rice bran as a by-product of the milling process is the hard outer brown layer of rice that possesses about 10 % of rice weight before discharging. This product contains fiber, fatty acid, starch, and proteins in addition to some phytochemicals with pharmaceutical properties [126]. Thanks to its different unsaturated fatty acids, rice bran could perform as an antioxidant and anti-cancer agent [127]. Still, unsaturated double bonds in its structure make it susceptible to oxidation and decay, necessitating vesicular encapsulation like niosomes. Manosroi et al. prepared rice bran niosomal encapsulation using non-heated supercritical carbon dioxide (scCO_2_) for anti-hair loss applications [127]. Lycopene is a red tetraterpenoid hydrocarbon found in some vegetables and red fruits such as tomatoes. It is known for its anti-oxidant activities which can help combat diabetes, cardiovascular diseases, and cancer [128]. Due to its susceptibility to heat, light, and biological oxidants caused by its unsaturated bonds, protection techniques like encapsulating by niosome nanoparticles have been used [129]. Sharma et al. reported niosomal encapsulation of lycopene by adsorption-hydration method to investigate its anti-diabetic and anti-cancer applications [130]. The zingiber belongs to the *Zingiberaceae* family and is a plant native to Southeast Asia. It contains anti-inflammatory, anti-pain, and anti-histamine Plai oil which could be used as medicinal phytochemicals [131]. The extracted oil has low stability upon exposure to air or light due to rapid Physico-chemical alterations resulting in active site destruction [131]. Niosomal entrapment has been used as a protection technique for its stable delivery [131]. As a natural polyphenol containing unsaturated dilactone, EA is a phytochemical that can be found in numerous vegetables and fruits. EA possesses antioxidant, anti-inflammatory, and skin-whitening properties. Its poor hydrophilic and weak solubility in organic solvents, however, require novel encapsulation techniques such as niosomes [132,133]. Junyaprasert et al. reported niosomal encapsulation of EA for dermal delivery with enhanced skin permeation [132].

Research in breast cancer treatment showed promising results in using phytofabricated nanocarriers alone or in conjunction with other loaded phytotherapeutics or chemotherapeutics. Moreover, a strong emphasis is placed on the anticancer pathways underlying the activity of phytochemicals since diverse mechanisms are implicated in their anticancer activity. Phytochemical and chemotherapeutic agents combined with nanotechnology might have extensive effects in the future [135]. Niosome as an applied nanocarrier has maximized the potential use of phytochemicals to reduce formulation challenges. Apart from improving solubility and stability, niosomes could prolong their half-life and even accomplish site-targeting delivery [135]. Extraordinary pharmaceutical properties of some phytochemicals combined with different niosomal encapsulations could be a promising novel approach for drug delivery purposes. Pharmaceutical applications of natural products-loaded niosomes are summarized in Table 3.

**Table 3:** Natural products loaded niosomes for pharmaceutical applications.

Niosome Type	Natural Product	Production Method	Size (nm)	EE (%)	Disease Type	Products	*In-vivo*/*In-vitro*	Results	Ref
LUV	Rice Bran	scCO_2_	480.9 ± 270.8	47.54–64.47	Skin Aging	Anti-aging effects in gel and cream	Rabit Skin/Fibroblast cells	Skin lightning, thickness, roughness and elasticity improvement	[126]
LUV	Lycopene	Adsorption-Hydration	175–235	62.8 ± 2	Diabetes	Anti-Diabetic vesicles	Wistar rat	Efficient delivery.	[130]
								Blood glucose level reduction	
LUV	Lycopene	Adsorption-Hydration	170–230	62.76 ± 2	Cervical and breast Cancer	Lycopene encapsulation	MCF-7 and HeLa cells, Wistar rat	Anti-cancer activity	[137]
SUV	Zingiber	Film hydration	100	–	Inflammation		Wistar rat	Antioxidant and anti-inflammatory	[131]
LUV	Turmeric	Transmembrane pH gradient	400–500	81.69	Mosquito vectors		Against larvae	Mortality of larvae	[138]
LUV	Embelin	Thin film hydration	500–700	63.32–80.00	Diabetes		Wistar rat	Hypoglycemic activity	[118]
LUV	Gymnema sylvestre	Thin film hydration	229.5 ± 30	57.8–85.3	Diabetes	Anti-Diabetic vesicles	Wistar rat		[139]
LUV	Silymarin	Hand Shaking	256.2–541.1	43.8–70.61		Hepatoprtective	Wistar rat	Increasing drug bioavailability	[140]
LUV	Ellagic acid	Reverse phase evaporation	124–1776	1.35–26.75			Human skin & Franz diffusion cell	Efficient delivery of EA through epidermis	[141]
LUV	Ammonium Glycyrrhizinate	Thin film hydration and high-speed stirring	363–622	13.2–40	Eczema and psoriasis	Dermal administration	–		[142]
LUV	Resveratrol	Thin film hydration	139–227	16.8–72.5	–	Yoghurt additive	–	–	[143]
SUV and LUV	Nerium oleander	Thin film hydration	59.1–334.0	13.24–16.20			Alveolar type-II and cervical cancer cell	Antioxidant activity	[144]
LUV	Curcumin	Thin film hydration	250 ± 20	90	Cancer	Anticancer, anti-tubercular and anti-inflammatory	HeLa	Multi-drug delivery enhancement	[29]
LUV	Morusin	Thin film hydration	479	97	Cancer	Anticancer therapy	MDA-MB-453, HT-29, PANC-1, SKOV-3, and L929		[145]
SUV	Curcumin	Thin film and pH-gradient	48–185.9	50.21–95.11	Cancer	Anticancer therapy	Saos-2, MG-63, and KG-1	Multi hydrophilic and hydrophobic drug delivery	[119]
LUV	Papain	Thin film hydration and sonication	220.7–520.2	–	Scar of skin	Skin treatment	Sprague-Dawley rats' skin	Skin permeation enhancement	[146]
LUV	Gambogenic acid	Ethanol injection	98.3–299.7	36.09–68.20	Cancer	Anticancer drug candidate	–	Effective release and increasing duration	[22]
SUV	Curcumin	Thin film hydration	101.5–125.1	52.24–85.42	Cancer	Anticancer therapy	MCF-7, MCF-10A	Improving therapeutic effectiveness of cancer treatments	[121]
LUV	Green tea	Thin film hydration	338.3	56.39–77.80	Skin Protection	Antioxidants niosomal gel	–	Efficient encapsulation	[147]
LUV	Resveratrol	Thin film hydration and ethanol injection	284 ± 28–496 ± 39	15±1–48 ± 3	Skin inflammation and irritation	Topical use and delivery	Newborn Pig skin		[125]
SUV	Morin	Thin film hydration	109–233	55.47–78.94	Cancer and Parkinson	Antioxidant and anticancer activity in brain	Wistar rat	Improvement in AUC of MH	[148]
MLV	Ellagic acid	Reverse phase evaporation	312–560	25.63–38.73	–	Dermal delivery system	Human skin	Permeation enhancement to the skin	[132]
LUV	Aloe vera	Reverse phase evaporation	270.08	42.04	Skin defects and wounds	Skin wound dressing	Fibroblast cell	Accelerating healing process	[149]
–	Silibinin	Reverse phase evaporation	–	49.50–86.38	Cancer	Anti-tumoural activity	T47D cell	Altering level of miRNA expression	[150]
–	Resveratrol	Thin film hydration	–	97.00 ± 0.02	–	Hydrogel systems encapsulating niosomes	–	Preventing *trans*-to-cis photoisomerization of resveratrol	[123]
MLV and SUV	Resveratrol	Mechanical agitation and sonication	200–900	<40.96	Antioxidant	Functional food	–		[151]
SUV	Rice Bran	chloroform film method with sonication	44.86–75.67	–	–	Anti-hair loss products	Fibroblast cell		[127]
LUV	Ginkgo biloba	Film dispersion–homogenization	140.9–505.1	38.7–57.7	Auto-oxidation, antitumor, protective in nervous system	Delivery of GbE to the brain	Wistar rat		[152]
–	Curcumin	Ether injection	–	–	–	Transdermal formulation gel	Wistar rat skin	–	[122]
LUV	Artemisia absinthium	Thin film hydration	174 ± 2.56	66.73	Amyloid aggregation		–		[153]
SUV	Marigold	Thin film hydration	57.61 ± 25.27 and 82.72 ± 21.92	59	Cytotoxicity, wound healing, antioxidant		Vero cell		[154]
LUV	Zingiber		690 and 1930	–		Anti-inflammatory	Newborn pigs skin and Male ICR mice	Chemical stability and skin permeation enhancement	[155]
LUV	Propolis	Reverse phase evaporation	237–333	91 ± 0.48	Oral recurrent aphthous ulcer	Antimicrobial compound	Franz diffusion cell		[156]
SUV	Carum	Thin film hydration	100–200	86.25–92.32	Cancer	Breast cancer treatment	MCF7	Novel high efficiency carrier	[157]

### 7.2 Gene delivery

**Fig. 5:**
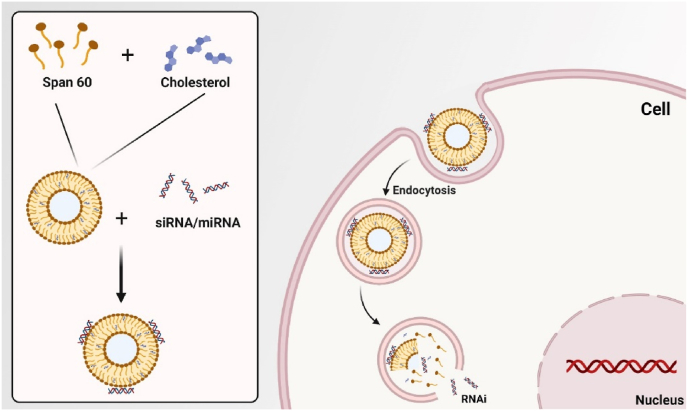
Niosomes designed for intracellular delivery of siRNA/miRNA and activatable labeling of cells upon dequenching, modified based on [166].

Gene therapy has been utilized as an effective technique in the treatment of hereditary human disorders using non-viral carriers to improve the cellular absorption characteristics of nucleic acids (Fig. 5). The properties of the vector significantly impact the effectiveness of gene therapy [158,159]. Even though niosomes have been present for almost three decades, only a few research have been conducted to investigate their potential as gene delivery vectors. Compared to liposomes, niosomes have higher storage and chemical stability due to the presence of non-ionic surfactants. These non-ionic surfactants also reduce the toxicity of niosomes as well as their fabrication cost. These features encourage research on the use of niosomes in gene delivery applications [160,161] (Table 4). In reported studies, niosomes have been employed as oligonucleotide carriers to treat various ailments. A strategy was demonstrated to transfer pCMS-eGFP plasmid to the retina using niosomes [120]. A cationic niosome formulation prepared with 2-di(tetradecoxy)propane-1-amine, squalene and polysorbate 80 was used for compact transport of a 5 kb-long pCMS-eGFP DNA plasmid in the eye. RPE cells were modestly transfected following the sub-retinal injection in rats, while GFP expression in the inner retinal layers was induced by intravitreal injection. While maintaining the transfection efficiency, the inclusion of protamine in the formulation enhanced nucleus targeting and allowed transfection of a small proportion of photoreceptor cells following sub-retinal injection [120]. It was also discovered that encapsulating genes encoding hepatitis B surface antigens (HBsAgs) in niosomes induced an immune response to produce blood antibodies and endogenous cytokines comparable to intramuscularly recombinant HBsAgs or topical liposomes [162]. Qtaish et al. developed a novel niosomal formulation with long-term biophysical stability for non-viral gene delivery to the retina [163]. Niosome as a non-viral vector has shown advantageous features for gene delivery, which include low toxicity, high stability, and easy production [161].

**Table 4:** Niosome-based non-viral vectors designed for gene delivery.

Components	Preparation method	Cargo	Application	Ref
Polysorbate 20 and 80, Poloxamer 407, DOTMA^c^, DTPA, Squalene, chloroquine	Oil-in-water emulsion	CFTR gene^a^	Treatment of human cystic fibrosis	[167]
DOTMA, Tween 20	Oil-in water emulsion	Sphingolipids	Treatment of retinal and brain diseases	[168]
DTPA^d^, DDAB^e^, DOTAP^f^, SQ, Tween, Polyoxymethylene, Alkyl ethers, Span	Thin-film	ASO^b^	–	[159]
Polysorbate 80, Poloxamer 407	Reverse-phase evaporation	pCMS-EGFP plasmid	Delivering to urine-derived mesenchymal stem cells	[169]
DDAB, Tween 85 and 20	Microfluidic	siRNA GFP	Chemotherapy	[170]
DOTAP, Span 80, DOPE^g^, TPGS^h^	Ethanol injection	siRNA and miRNA	Chemotherapy	[165]
CTAB^i^, ergosterol, Fe_3_O_4_@SiO_2_	Thin-film	Pm-cherry-N1	Negative and positive trigger in HEK-293 cell line	[171]
DTPA, polysorbate 80	Reverse-phase evaporation	pUNO1-hBMP-7	Bone regeneration	[172]

a The cystic fibrosis transmembrane conductance regulator.

b Antisense Oligonucleotide.

c N-[1-(2,3-dioleoyloxy) propyl]-N,N,N-trimethylammonium methyl sulfate.

d Diethylenetriamine pentaacetate.

e Didodecyldimethylammonium bromide.

f 1,2-Dioleoyl-3-trimethylammonium propane.

g Dioleoyl phosphatidylethanolamine.

h Tocopheryl Polyethylene Glycol Succinate.

i Cetyl Trimethyl Ammonium Bromide.

Niosomes can be potentially used as a delivery vehicle for stem cells. A study on niosomes revealed their applicability in the delivery of RNAs to human mesenchymal stem cells (hMSC) to promote cell differentiation [164]. Niosomes based on cationic lipid 1-(2 dimethyl aminoethyl)-3-[2,3-di (tetradecoxy) propyl] urea and paired with squalene as a “helper” lipid and polysorbate 80 were recently prepared, which showed the capability to transfect the rat cerebral cortex upon in situ delivery [165]. The solvent evaporation method was utilized to create cationic niosomes with a diameter of 200 nm, a low polydispersity index (PDI) value (0.21), and a positive surface charge of over 30 mV. Their physicochemical characteristics remained stable after 100 days of storage at 4 °C. The resultant nioplexes were able to transfect both neurons and nonneuronal cells in primary cultures, taken from the brain of rat embryos [161].

### 7.3 Cancer treatment

Niosomal formulations can deliver various anticancer drugs with low side effects. Conventional chemotherapy cannot selectively target the cancerous cells and is associated with low therapeutic efficacy and a high incidence of side effects and toxicity to normal cells. Colloidal niosomal formulations are promising systems for drug delivery to cancerous tissues, passively and actively. Delivery of anticancer drugs by niosomal formulations can overcome low bioavailability and stability, significant risk of side effects, and inadequate access to the drug because of low permeation of the blood-brain barrier. The niosomal formulations have been reported to decrease the toxicity of Withaferin–A (WA) as an active constituent of *Withania somnifera* [173], tamoxifen (TMX)/curcumin [174], and curcumin [112]. Niosomes use different release mechanisms in cancer tissues or cells. Various stimuli, including temperature, light, pH, enzymatic decomposition, and ultrasound have been employed to activate the decomposition of bilayer vesicles [175]. Sharafshadeh et al. developed a formulation of alginate-coated niosome-based nanocarriers for the co-delivery of doxorubicin (Dox) and cisplatin (Cis) for the treatment of breast and ovarian cancers. Results proved the synergetic cell proliferation inhibitory impacts of Cis and Dox against MCF-7 and A2780 cancer cells.

**Fig. 6:**
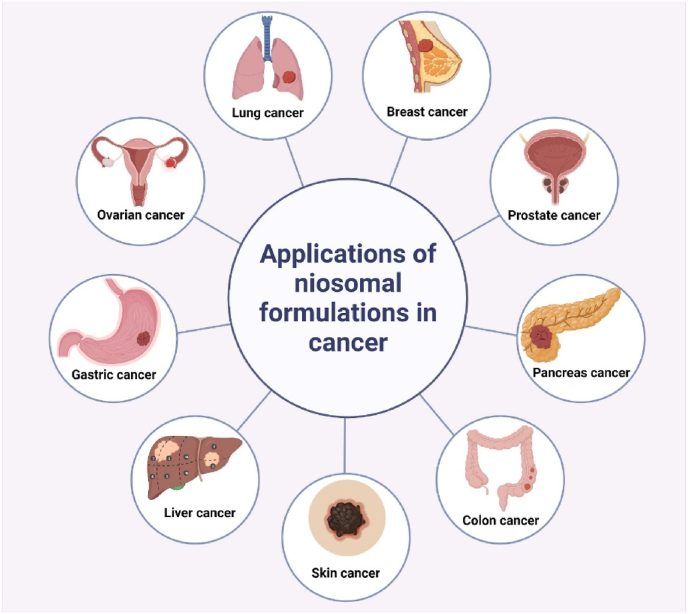
Applications of niosomal formulations in treatment of various cancer diseases.

The efficiency of niosomal formulation for ovarian and breast cancer treatment was explored [176]. Zarepour et al. prepared a new nano-drug delivery platform for the treatment of lung cancer, using niosomal formulation consisting of curcumin coated with a chitosan polymeric shell, alongside Rose Bengal (RB) as a photosensitizer with antibacterial properties. They showed great antibacterial and anticancer effects against Gram-negative bacteria and lung cancer cells [177]. Saharkhiz et al. developed a novel formulation consisting of doxorubicin-loaded pH-responsive stealth niosomes and CdSe/ZnS Quantum dots as an imaging agent. eThis new nanoformulation showed potential for future cancer theranostic applications [178]. Various niosomal formulations allowed a greater reduction in the expression of genes involved in metastasis including COL10A1, MMP2, and MMP9 [179]. Moreover, the niosomal nanoparticles showed high anti-proliferative potential by restraining anti-apoptotic and inducing apoptotic gene expression in A549 lung cancer cells [180]. In the following sections, *in-vitro* and *in-vivo* studies of recent works on anticancer drug-loaded niosomes have been examined, and some of the remarkable outcomes achieved in those works have been detailed (Fig. 6).

#### 7.3.1 In-vitro study

Various *in vitro* studies have been carried out to use niosomes for cancer treatment. Hemati et al. prepared and optimized the cationic PEGylated niosome loaded with anti-cancer drugs and siRNA to develop the therapeutic response [181]. Hydrophilic Dox and hydrophobic QC (Quercetin) were loaded within the nanocarrier and lipid layers. Moreover, siRNA was loaded on cationic PEGylated niosomes. An optimized formulation can perform passive targeting depending on temperatures and pH of normal and cancer cells. As a result, optimized cationic PEGylated niosomes could offer an efficient delivery system for triple-combination therapy with enhanced therapeutic efficiency [181]. Shah et al. developed a niosomal formulation for the delivery of Gamma oryzanol (OZ, an anti-cancer agent) as a natural antioxidant with skin anti-aging features [182]. Niosomal formulation of OZ can resolve its inadequate aqueous solvability and restricted permeability. This niosomal gel could be used as a prophylactic skin cancer treatment. This study showed the superiority of the niosomal formulation in terms of drug retention, which is important for long-term delivery [182]. Saimi et al. developed a niosomal nanocarrier to deliver Gemcitabine (Gem) and Cisplatin (Cis) for lung cancer treatment. Gem and Cis have high toxicity in high dosages. An optimized low-dosage niosomal formulation of Gem and Cis (NGC) showed excellent potential in aerosolized delivery systems to treat lung cancer, which requires further *in-vivo* assessments [183]. Hu et al. developed a vesicle system targeting hepatocellular carcinoma which rapidly released the drug tanshinone IIA into the tumor cell [184]. Pharmacokinetic experiments confirmed that the niosomal formulation can significantly prolong blood circulation. The developed niosomes are expected to be a safe and effective drug delivery system for the treatment of liver cancer. Kassem et al. prepared an imatinib mesylate (IM)-loaded niosomes to enhance its chemotherapeutic efficiency and selectivity toward cancer cells, suggesting promising effectiveness in combating cancer [185]. Niosomal IM formulation exhibited high cytotoxicity against colon cancer cell line (HCT-116), breast cancer cell line (MCF-7), or hepatocellular carcinoma (HepG2) [185]. In another work, Akbarzadeh et al. developed a niosomal formulation with antibacterial and anti-cancer activities for doxycycline delivery [186]. The niosomal formulation had a great encapsulation efficiency (EE) and showed a significant decrement in minimal inhibition concentration (MIC) values against various Gram-negative and Gram-positive bacteria. Doxycycline-loaded niosomes have chemotherapy effects on prostate cancer cells (PC3) while exhibiting biocompatibility toward normal HEK293 cells [187]. Obeid et al. encapsulated balanocarpol into niosomes nanoparticles and found that niosomal formulation reduced toxicity and enhanced the solubility of balanocarpol [175]. This niosomal formulation can be used for developing an efficient balanocarpol-based anticancer remedy. Zare-Zardini et al. characterized a novel niosomal formulation containing ginsenoside Rh2, an anticancer agent, for increased antitumor efficacy in prostate cancer [188]. Akbarzadeh et al. optimized niosomal formulation for curcumin delivery as a natural chemical compound for antitumor therapies [112]. As mentioned above, insufficient stability, low solubility, and rapid degradability hinder the clinical use of curcumin. Curcumin-niosomes can improve cellular uptake, cytotoxic effects, cell cycle detention, and apoptotic activities in ovarian cancer A2780 cells [189]. Maniam et al. synthesized and optimized a niosomal formulation for dual-drug delivery in pancreatic cancer cells *in-vitro*. Niosomal formulation managed in co-encapsulating Gem and tocotrienols with a nine-fold improvement in cytotoxicity of the combination, supported by significantly higher cellular uptake of Gem in the cells [190]. Some anticancer drug-loaded niosomal formulations, type of cancer, their characterization, and outcome are listed in Table 5.

**Table 5:** Anticancer niosomal formulations. PDI: polydispersity index, EE: encapsulation efficiency.

Type of cancer	Study		Characterization	Outcome	Ref
Breast cancer	Balanocarpol, as a potential anticancer drug encapsulated into niosome and evaluated against human breast and ovarian cancer cell lines		Particles size: ∼175 nm, PDI: 0.088, EE: ∼40 %	Balanocarpol showed superior anticancer effect over the free compound when tested *in-vitro* on human ovarian carcinoma (A2780) and human breast carcinoma (ZR-75-1)	[175]
Prostate cancer	Nano niosomal formulation encapsulated Ginsenoside Rh2 for developed antitumor effectiveness and assessment *in-vitro*		The mean size, PDI, zeta potential, and EE of 93.5 ± 2.1 nm, 0.203 ± 0.01, +4.65 ± 0.65, and 98.32 ± 2.4 %, respectively.	The cellular uptake and cytotoxic activity enhanced with niosomal delivery of the Ginsenoside Rh2	[188]
Liver cancer	Developed galactose-modified pH-sensitive niosomal formulation for target delivery of Tanshinone IIA		Particle size: ∼53.72 nm, Zeta potential: −28.31 mV, EE: 84.70 %	An optimized niosomal formulation showed a safe and efficient drug delivery for liver cancer therapy.	[184]
Prostate cancer	Prepared an optimized doxycycline-loaded niosomal formulation for treatment of infection-associated prostate cancer		Particle average diameter: 254 ± 8 nm,	The niosomal formulation regulated the drug release and showed a slower and delayed-release at physiological pH.	[187]
Colon cancer	Optimized imatinib mesylate-loaded niosomes for human colon adenocarcinoma		Particle size: 391.18–574.92 nm, PDI: v 0.126–0.498, EE: 71.4 %–85.9 %	Niosomal formulation developed imatinib mesylate (IM) efficiency and selectivity toward cancer cells	[185]
Lung cancer	Prepared and optimized and *in-vitro* evaluation of aerosolized niosomal formulation including Gem and Cis for lung cancer treatment		Particle size, PDI, Zeta potential, and EE of 166.45 nm, 0.16, −15.28 mV, 96.22 %, respectively.	The results indicated that the optimized niosomal formulation has great potential in aerosolized delivery systems to treat lung cancer.	[183]
Skin cancer	Preparation and optimization of the OZ niosomal formulation for skin cancer		Average vesicle size: 196.6 nm, EE: 78.31 %	OZ niosomal gel can be used f as a prophylactic treatment for skin cancer. The prototype was developed.	[182]
Ovarian cancer	Prepared and optimized niosomal formulation for encapsulation of curcumin and characterized its cytotoxic effect on ovarian cancer cells	Average vesicle size: 84.15 ± 4.03 nm, EE: 92.3 ± 0.4 %		Niosomal formulation showed developed cytotoxic activity and apoptotic rate of ovarian cancer A2780 cells.	[189]
Pancreatic cancer	Prepared and optimized niosomal formulation for co-encapsulation of gemcitabine and tocotrienols that developed efficiency in pancreatic cancer treatment	Vesicle size: 161.9 ± 0.5 nm, EE: 20.07 ± 0.22 % for Gem and 34.52 ± 0.10 % for tocotrienols		The study demonstrated the synthesis of dual drug niosomes and their efficiency on pancreatic cancer cells *in-vitro*	[190]
Gastric/Prostate/Breast cancers	Synthesized an innovative niosomal formulation containing Dox, QC and siRNA for cancer Treatment		Vesicle size and Zeta potential of 52.8 ± 2.7 nm and +27.4 ± 2.3 mV, respectively. EE of 86.4 ± 2.1 % for Dox and 94.9 ± 3.9 % for QC	Niosomal formulation for co-delivery of drugs and siRNA showed a developed anti-cancer activity against the tumor cell death.	[181]

#### 7.3.2 In-vivo study

In-depth *in-vivo* studies have been conducted to evaluate the efficacy of niosome nanocarriers in various therapeutic applications. Shah et al. developed an anticancer niosomal formulation containing Withaferin-A (WA), a bioactive compound from Withania somnifera, and subsequently assessed its delivery to cancer cells in an *in-vivo* study using mice. The results demonstrated significant tumor volume reductions of 71 % and 51 % in the cisplatin and WA niosomal formulation treatment groups, respectively. These findings corroborate the potential of nanoniosome formulations to elicit favorable responses in diverse diseases. Furthermore, molecular modeling indicated the formation of a stable complex with WA, characterized by stable hydrophobic contacts, facilitating controlled drug release properties of the formulation [173].

In another investigation, Ghadi et al. formulated curcumin and encapsulated it in niosomal hyaluronan. The *in-vivo* assessment, conducted in rats, revealed that this formulation incorporating a polymeric niosomal structure of hyaluronan enhanced effectiveness in co-delivering natural hydrophobic products and the anti-inflammatory efficacy. Moreover, the effect of the basic curcumin suspension diminished within 24 h, while curcumin-loaded niosomes exhibited sustained effects for the same duration, highlighting the stability and efficiency of this niosomal formulation for oral administration [191]. It was also reported that a lysine-mediated niosomal formulation exhibited high efficacy and low toxicity, effectively targeting and eliminating cancer cells *in-vivo* mice study [192].

In the study conducted by Barani et al., pH-responsive niosomes loaded with paclitaxel (PTX) and modified with ergosterol were evaluated for their anticancer effects *in-vivo*, using male adult Sprague-Dawley rats. The results demonstrated enhanced therapeutic efficacy of PTX when encapsulated in the niosomal formulation, with lower toxicity to healthy cells compared to free PTX. Notably, both free PTX and niosomal PTX exhibited dose-dependent toxic effects on the liver and kidneys of rats, but niosomal PTX displayed fewer side effects. Additionally, histopathological analyses revealed the ability of both free PTX and niosomal PTX to penetrate liver and kidney tissues [193].

Salem et al. utilized pH-sensitive Triaryl-(Z)-olefin (TZO) niosomes for the treatment of breast cancer. The TZO-loaded niosomal formulations were synthesized with various concentrations of chitosan and Glyceryl monooleate (GCM). *In-vivo* assessments demonstrated significant tumor regression and TZO localization for the optimized formulation. This study highlighted the potential of niosomal formulations in enhancing therapeutic outcomes while reducing side effects [195,].

Moghaddam et al. developed a niosomal formulation of melittin, a component of honey bee venom, and assessed its anti-cancer effects against breast cancer in *in-vivo* experiments using mice. This study revealed superior anti-cancer activities of melittin-loaded niosomes compared to free melittin, offering a promising and efficient treatment for breast cancer with fewer side effects. The *in-vivo* experiments indicated the ability of melittin-loaded niosomes to drain angiogenic growth factors and improve vascular supply, showcasing the potential of nanoscale carriers for targeted delivery of cytolytic peptides to solid tumors [197].

Sabry et al. designed a delivery system for Galangin, a flavonoid with anti-tumor activity but poor solubility and bioavailability. *In-vivo* assessments demonstrated that Galangin-loaded niosomal formulations effectively reduced serum levels of liver biomarkers and showed hepatic foci reduction, indicating their potential in reducing neoplastic liver lesions [198,]. This suggests that optimized niosomal formulations could serve as targeted systems to enhance antitumor activity against liver cancer.

Barani reported a pH-responsive methotrexate (MTX) niosomal formulation modified with ergosterol for cancer treatment. *In-vivo* studies showed that niosome formulation improved the solubility of MTX, displayed enhanced anticancer activity in tumor-bearing mice. The results suggested that niosomal MTX may have limited toxic effects at multiple doses, highlighting its potential as an effective drug delivery system [200].

In summary, the *in-vivo* assessments of developed niosomes loaded with anticancer therapeutics have shown promising results in terms of improving drug solubility, enhancing therapeutic efficacy, and reducing side effects. These studies have demonstrated the potential of niosomal formulations as effective nanocarriers for various cancer treatments. Researchers continue to explore the diverse applications of niosomes based on their types, morphological properties, formulations, fabrication methods, and surface functionalization strategies, emphasizing the need for a comprehensive review of their applications in drug delivery [See Table 6 for a summary of *in-vivo* assessments of niosome-loaded anticancer therapeutics].

**Table 6:** Anticancer drug-loaded niosomal formulations, *in-vivo* models, their characterization, and the outcome.

Study	Characterization	Outcome	Route of administration	Ref
Co-delivery of hydrophobic natural products	Size: 260.37 ± 6.58 nm	Enhancing the stability of curcumin and QC and their pharmacological efficacy. Better anti-inflammatory impact	Orally and Subcutaneous injection	[193]
	PDI: 0.42 ± 0.03			
	Zeta potential: −34.97 ± 1.5 mv			
	EE Curcumin: 98.85 ± 0.55 %			
	EE QC: 93.13 ± 1.22 %			
Synthesized and characterization of anticancer niosomal withaferin–A formulation	Size: 278 ± 5 nm	A significant antitumor effect of WA-niosomes *in-vivo* was discovered as a prototype of cancer	Injected intraperitoneally into the flanks of the test animal	[173]
	EE: 87 ± 3 %			
	Zeta Potential: −41.72 ± 6.01 mV			
	PDI: 0.419 ± 0.073			
Curcumin entrapped hyaluronan containing niosomes	Size: 249.83 ± 6.38 nm	The anti-inflammatory effect of the hyaluronan containing niosomes was higher than free curcumin	Orally and subcutaneous injection	[201]
	EE: 98.28 ± 0.278 % (w/w)			
	PDI: 0.36 ± 0.04			
	Zeta potential: −34.83 ± 0.5 mv			
Treatment of breast cancer with engineered novel pH-sensitive Triaryl-(Z)-olefin niosomes containing hydrogel	Size: 325.5 ± 9.53 nm,	Significant antitumor effect shown compared to TMX	Intra-tumour injection	[195]
	EE: 91.18 ± 0.72 % with slow drug release of 45.41 ± 1.20 % within 8 h.			
Smart stimuli-responsive biofunctionalized niosomal nanocarriers for programmed release of bioactive compounds into cancer cells	Size: 163.27 nm zeta potential: −0.71 mV	Delivery to cancer models caused a higher tumor inhibition rate than in other groups.	Subcutaneous injection	[192]
Delivery of vinblastine-containing niosomes resulted in potent cytotoxicity on tumor cells	Size: 234.3 ± 11.4 nm zeta potential: −34.6 ± 4.2 mV	In animal model, PnVB exhibited stronger tumor inhibitory effect and longer life time in comparison to free VB	Administered intravenously (tail vein) and inoculated subcutaneously into the right flank	[202]
	EE: 99.92 ± 1.6 %			
Paclitaxel (PTX)-loaded pH-responsive niosomes modified with ergosterol were developed	Size: 240 nm	Encapsulating PTX in niosomal formulation developed its therapeutic efficacy	Intraperitoneally injected	[193]
	EE: 77 %			
Delivery of melittin-loaded niosomes for breast cancer treatment	Size: 121.4 nm	Melittin-loaded niosome enhanced targeting, encapsulation efficiency, PDI, and release rate and shows a high anticancer effect on cell lines	Intraperitoneally injected	[186]
	PDI: 0.211			
	EE:79.32 %			
Prepared and characterized *in-vitro* and *in-vivo* niosomal formulation loaded with Galangin on chemically induced hepatocellular carcinoma	Size: 173.7–355.6 nm,	Histopathological and immunohistochemical examinations revealed that GAL-loaded niosomes allowed a meaningful decrease in MCM3 immunostained hepatocytes and liver tumor lesions with few liver adenomas	Subcutaneous injection	[198]
	EE: 45.13 %–77.69 %,			
	Drug loading capacity (DL%): 9.02 %–16.72 %			

#### 7.3.3 Niosome nanocarrier in clinical trial

Up to now, only a limited number of studies have progressed to clinical trials in the context of niosome nanocarriers, despite the extensive research and numerous investigations into niosome formulations conducted over the past few decades. Much of the clinical research primarily centered on topical administration.

An examination of recent findings from the last five years reveals that incorporating drugs into niosomes has demonstrated enhanced therapeutic efficacy and reduced side effects. For instance, Mohammadi et al. conducted a clinical study comparing the effectiveness of isotretinoin 0.05 % niosomal gel with adapalene 0.1 % gel in treating acne vulgaris. The results indicated that the clinical responses for comedones and inflammatory lesions were 68 % and 79 % in the isotretinoin 0.05 % niosomal gel group, as opposed to 65 % and 76 % in the adapalene gel group. This data suggests that isotretinoin 0.05 % niosomal gel exhibits slightly fewer side effects and greater effectiveness in treating acne vulgaris compared to adapalene 0.1 % gel [203].

Furthermore, in a clinical trial conducted by Farajzadeh et al., the effectiveness of intralesional Glucantime combined with niosomal zinc sulfate was compared to intralesional Glucantime combined with cryotherapy in treating acute cutaneous leishmaniasis. Patients were divided into two groups, A and B. Group A received weekly intralesional meglumine antimonite and twice-daily niosomal topical zinc sulfate, while group B received weekly intralesional Glucantime and cryotherapy every other week. The results showed that the incomplete response rate was 16.6 % in group A and 12.9 % in group B, while the total response rate was 73.3 % in group A and 80.6 % in group B. These findings suggest that niosomal zinc sulfate, in combination with intralesional Glucantime, exhibits similar efficacy to cryotherapy with intralesional Glucantime in treating acute cutaneous leishmaniasis [204].

Additionally, Damrongrungruang et al. conducted a clinical study in which they investigated anthocyanin complex (AC) niosomal gels. A randomized block placebo-controlled double-blind clinical trial involving 60 volunteers with oral wounds demonstrated that AC niosome gel accelerated wound closure, reduced pain associated with oral wounds, and improved the participants' quality of life more effectively than AC gel, triamcinolone gel, and placebo gel, highlighting the therapeutic potential of AC niosomes [205].

In the realm of clinical trial research, there is a need for further efforts and proactive approaches to harness the potential of niosomes as nanocarriers. The limited number of studies available for clinical trials hinders technological advancements. Additionally, regulatory challenges have arisen due to the complexity of implementing nanocarriers in drug delivery. To effectively utilize applied nanotechnology in this field, clinical trial settings and an efficient regulatory framework are essential.

## 8 Limitations and challenges in the development of niosomes

Niosomes have received a great deal of attention as promising drug delivery nanosystems for the administration of various therapeutics, such as natural products and anticancer drugs, by the aforementioned methods into the body. In recent years, niosomes have been introduced as an inexpensive and stable alternative to ordinary and conventional nanocarriers. Nearly all types of drug delivery systems can be developed in the form of niosomal aqueous suspensions. Their shape, size, and entrapment efficiency can be easily altered by modifying the different parameters mentioned above. Their ability to encapsulate and deliver both hydrophilic and hydrophobic therapeutics distinguishes them from other drug-delivery vehicles. Also, the potential for scaling up and low-cost production makes niosomes an interesting nanosystem for industrial pharmaceutics, particularly for the cosmetic industry. It is worth mentioning that the self-assembly of non-ionic surfactants provides a chemically and physically stable structure, compared to other nanocarriers, like liposomes. Excellent chemical stability, osmotic activity, and prolonged durability are among the major superiorities of niosomes over liposomes. Thanks to the presence of a hydrophilic functional group on their head, the surface of the niosomes can be comfortably formed and changed. Niosomes also have less toxicity and more compatibility and degradability in biological systems compared to liposomes [5,24,75,80].

Despite the aforementioned advantages, niosomal delivery systems suffer from some serious challenges. The major hindrance that poses an obstacle in the utilization of niosomes in the drug delivery field is sterilization. The proposed methods for synthesis of niosomes are performed under septic conditions. Heat and steam sterilization strategies could be destructive to surfactants and lipids in the molecular structure of niosomes and cause drug leakage due to the disruption of the bilayer membrane. Researchers have proposed membrane filtration as a solution for this issue; however, the used filter would not be useful for niosomes with a size greater than 200 nm [206]. Gamma radiation is the other strategy to address the sterilization problem of niosomes. This is a crude method since the effect of the radiation on the physicochemical properties of niosomes has not been fully investigated. The other challenge of niosomes that needs to be addressed is the toxicity of the non-ionic-based formulation of this nanocarrier [207]. Some reports confirm the toxicity of surfactants; however, there is a dearth of researchers focused on investigating the cytotoxicity of niosomes. In one study, Hofland et al. examined the physicochemical characteristics of non-ionic surfactants. According to the results, the bonding between the polyoxyethylene head group and the alkyl chain has harmful effects on cell proliferation [208]. In a recent study conducted by Abdelkader et al., the toxicity of Span 60-based niosome has been investigated. The study disclosed that niosomes pose minimal conjunctival and corneal irritation, and they have acceptable ocular tolerability [209]. Nevertheless, there is a research gap in investigating the toxicity of niosome and its components after *in-vivo* administration.

Although niosomes have been utilized for various purposes, they need further developments to extend their applications in nanomedicine. For example, providing a sterile fabrication method and conducting a comprehensive *in-vivo* investigation of the toxicity of niosomes are imperative for mecidal applications. According to the ongoing works on niosomes, it seems that more research is needed to explore the synthesis and use of amphiphilic molecules as building blocks of niosomes. These molecules with a duality of functions could provide biologically active structures and give a targeting function to niosomes [210]. Moreover, there is a need for the decoration of niosomes with brain-specific ligands, such as chlorotoxin and glucosamine, to improve the permeability and penetration of functionalized niosomes loaded with drugs across the blood-brain barrier. Imparting the advantages of niosomes for scale-up production can broaden their applications in the pharmaceutical industry, particularly in the cosmetic one. For this purpose, developing a simple and low-cost production method for cosmeceutical products would be appreciated. The proposed method should provide certain benefits, such as enhanced elasticity, improved skin penetration, and better stability and deformability. Due to the outstanding results of using niosomes for skin therapy, any work on developing niosomes and their production methods would be appreciated in the cosmetic industry. According to the aforementioned approaches for developing niosomes as future studies, niosomes with proposed developments would be an ideal solution for miscellaneous industrial and research issues in the realm of pharmacy. In clinical trial research, additional attempts and efforts are necessary to surf the innovation influence niosome, and overcome the present regulatory challenges.

## 9 Niosome and other nanocarriers

The development of various nanocarriers for drug delivery has become more and more important in the pharmaceutical and cosmetic industry. Different nanoparticles such as lipid-based carriers (e.g. liposomes, micelles, emulsions, solid-lipid nanoparticles, and cell membranes), polymeric nanoparticles like dendrimers, and others have been introduced [1,4]. These nanocarriers have been synthesized and used widely for different goals by carrying various types of drugs toward different target organs. Although some of them have fabulous results in certain special cases, they have limitations in other situations [213,214]. Dendrimers as polymeric nanoparticles can be useed for drug delivery due to their nanometric size (e. g. 1–9 nm for 1–8 G polyamidoamine (PAMAM) [215], excellent monodispersity, available functional groups, and structural controllability ([216]) with high stability and water solubility [217,218]. The mentioned attractive features introduce dendrimers as one of the best candidates for drug delivery, however, the existing periphery charges in dendrimers can cause interaction with negatively charged cell membranes or other organelles such as nucleus, mitochondria, and enzymes, resulting in toxicity and cell lysis. Dendrimers with dense periphery charges can disrupt cell membrane, leading to intracellular elements leakage and killing cells [218]. PAMAM and polypropylene imine (PPI) as the most two common dendrimers with a positively charged periphery of amine-termination [219] are more toxic than negatively charged ones and this toxicity increases in higher generated dendrimers [220]. Although some enhancements are applied to these nanocarriers, more explorations and investigations are needed. Between different approaches, periphery modification by neutral and negatively charged groups is the most common. Also, some surface engineering methods same as biocompatible ligands such as PEG, carbohydrates, amino acids, and peptides have been used that not only decreased toxicity but also improved stability, drug leakage reduction, and increased solubility of hydrophobic drugs [221,222].

Lipid-based carriers as one of the main categories among the nanocarriers but they have limitations in certain applications. For example, solid lipid nanoparticles have excellent responses in encapsulating lipophilic drugs, but they are not faultless in hydrophilic drug encapsulation [223]. Scientists applied different modifications in solid lipid carriers and improved their features by conjugating with other polymeric and surfactant elements to enhance encapsulating hydrophilic drugs. Although these efforts were successful in some cases, they may change drug properties or even make them toxic [223,224].

Niosome as one of the youngest members in nanocarriers could have some benefits over the others in some circumstances. Niosome in comparison with liposome, the most attractive candid among the other lipid-based carriers, has lower toxicity, lower cost by easier fabrication, and more biocompatibility [225]. Micelles as another drug carrier with small size (<50 nm) can penetrate effectively through target organs like a cancerous colony and have high proficiency in cell internalization. However, they have a considerable stability problem that limit their usage for drug delivery. Micelles are often decomposed into free surfactants after dilution in physiological media such as blood. Thus, it may release carrying drugs before reaching the target site as the result of a burst disassembly, which can deteriorate the performance and cause harmfulness worries [226,227].

Cell membrane as a new category of nanocarriers has some advantages in comparison with other lipid-base and organic nanocarriers by offering cell-to-cell interactions and having functional elements on the surface including proteins making up living matters and mimicking nature [228,229]. Cell membranes encapsulating nanoparticles increase their biocompatibility by covering them with some lipids and carbohydrates that can be found in the body naturally. However, cell membranes have certain limitations for a wide usage in comparison with niosomes. First of all, the complexity of the membranes with their different functional proteins makes it impossible to synthesize them, and they are prepared based on decellularization and leakage of intracellular organelles [228,230]. Secondly, cell type and cell source (e.g. autologous or allogenic sources from the same patients) have considerable effects on their performance, adding more complexity index [228,231]. Moreover, producing autologous sources is time-consuming and cannot be used in some emergency cases. Furthermore, lacking a standardized and precise protocol for quality control, besides variations in each batch production, can have considerable uncertainty on final products. Other main concerns of having cell membranes as a qualified drug carrier are their purity when produced on a large scale [228,229], shorter half-life relative to liposomes [230], and instable at low temperatures during storage compared with niosomes [228].

## 10 Future perspective of niosome

Medical applications of nanocarriers are one of the most motivating fields among scientific areas of research. Niosome as a noble nanoparticle for drug delivery with relatively high safety and easy production and storage has attracted considerable attention for last years. Due to the vast potential in encapsulating various types of toxic, sensitive, and degradable drugs, allowing for effective targeting of specific organs without disrupting normal cell physiology and minimizing the side effects, it is expected that niosome will have a key role in the emerging medical treatments. Different niosomal formulations encapsulating a diverse array of drugs can be used for cancer therapy and tumor cells treatment, ensuring their continued prominence for the next decades. In the future, niosomes are likely to find a broad spectrum of applications due to their capacity to encapsulate both hydrophobic and hydrophilic substances and respond to pH changes.

## 11 Conclusion

Niosomes as self-assembly vesicles made by non-ionic surfactants are known as nanocarriers capable of controlled, sustained, and targeted drug delivery. Compared with various drug carriers, niosome nanoparticles were introduced as a high-potent novel drug carrier to overcome some disadvantages of the others. First of all, niosomal formulations, by offering aquatic and non-aquatic media, could be used for encapsulating both hydrophilic and hydrophobic drugs such as naturally-derived drugs, enzymes, peptides, genes, vaccines, anti-cancer drugs, and almost all types of pharmaceutical agents. Secondly, the non-ionic nature of the niosome makes it biocompatible. The advantages and capacities of niosomes, in comparison with other nanocarriers, such as their non-toxicity, higher chemical stability with a longer lifetime, easy modification by presenting functional groups to the surface, and useable by all types of administrations have increased their research interest, especially when the prospective of niosome can be improved through innovative arrangements, filling, and modification approaches. However, there are some issues related to niosomes requiring further research to make them clinically applicable, such as hydrolysis of the drugs in an aquatic suspension of niosome resulting in drug leakage or niosome aggregations. Further, several issues concerning niosomal encapsulated drugs need to be addressed, such as a large amount of drugs that remain unencapsulated during the synthesis, necessitating the streamlining of some preparation steps such as dialysis or filtrations. Moreover, cholesterol as a key component used in niosomal formulations providing higher stability may affect biological features of niosome membranes such as reducing its flexibility resulting in lower drug permeation inside it. Therefore, further studies are expected to improve niosomes properties to reveal new potentials and boost their translation.

## Credit authors statement

A.M., M.M.C., H·S., R.G., A.M., I.A., F·H., M.A., and F.M. Writing – original draft. Q.R. Conceptualization, Supervision, Writing – review & editing.

## Declaration of competing interest

The authors declare that they have no known competing financial interests or personal relationships that could have appeared to influence the work reported in this paper.

## Data Availability

No data was used for the research described in the article.
